# Prostate Imaging for Local Recurrence Reporting and Data System using Biparametric Magnetic Resonance Imaging: A Proposal

**DOI:** 10.5152/tud.2023.22228

**Published:** 2023-07-01

**Authors:** Michele Scialpi, Eugenio Martorana, Fabio Trippa, Alessandro Di Marzo, Giovanni Battista Scalera, Maria Cristina Aisa, Alfredo D’Andrea, Francesco Maria Mancioli, Refky Nicola, Pietro Scialpi, Aldo Di Blasi

**Affiliations:** 1 Department of Medicine and Surgery, Division of Diagnostic Imaging, Santa Maria della Misericordia Hospital, University of Perugia, Perugia, Italy; 2 Division of Urology, Nuovo Ospedale Civile Sassuolo, Modena, Italy; 3 Radiation Oncology Centre, Ospedale Santa Maria, Terni, Italy; 4 Division of Radiology, Perugia Hospital Authority, Perugia, Italy; 5 Department of Medicine and Surgery, Division of Obstetric and Gynaecology, Santa Maria della Misericordia Hospital, University of Perugia, Perugia, Italy; 6 Division of Radiology, Ospedale di Caserta, Caserta Italy; 7 Division of Radiology, Ospedale Santa Maria, Terni, Italy; 8 Department of Radiology, Syracuse, SUNY Upstate Medical, State University of New York Upstate Medical University, USA; 9 Division of Urology, Portogruaro Hospital, Venice, Italy; 10 Division of Radiology, Tivoli Hospital, Tivoli, Italy

**Keywords:** Prostate cancer (PCa), prostate cancer local recurrence (PCLR), radiation therapy (RT), radical prostatectomy (RP), magnetic resonance imaging, Prostate Magnetic Resonance Imaging for Local Recurrence Reporting (PI-RRADS)

## Abstract

We investigated a novel dedicated Prostate Imaging for Local Recurrence Reporting and Data System (PI-RRADS) in biochemical recurrence (BCR) after radiotherapy (RT) and radical prostatectomy (RP) evaluating biparametric magnetic resonance imaging (bpMRI) exams, at 3T MRI of 55 patients. Associating bpMRI and BCR data, we calculated bpMRI diagnostic accuracy. Four probability categories, from 1 (very low) to 4 (very high), were distinguished. In 20 patients with radiotherapy, 25% and 75% of lesions were reported as PI-RRADS 3, and 4, respectively. In 35 patients with radical prostatectomy, 7.7% of lesions were included in PI-RRADS 1-2, whereas 40.4% and 51.9% in PI-RRADS 3 and 4 categories, respectively. Excellent agreement and significant correlation between bpMRI and BCR were found. BpMRI showed sensitivity, specificity, positive predictive value, negative predictive value, false-positive value, false-negative value, and total diagnostic accuracy of 96.15%, 86.7%, 97.4 %, 81.25%, 13.3%, 3.8% and 94.6%, respectively. PI-RRADS based on bpMRI allows the detection and localization of local recurrence in BCR after RT and RP contributing in clinical management and treatment.

**Main Points**

- Biparametric magnetic resonance imaging is a valuable tool to detect prostate cancer local recurrence (PCLR) biochemical recurrence after radiotherapy and radical prostatectomy.
- Diffusion-weighted imaging/apparent diffusion coefficient detects PCLR as restricted diffusion area and T2-weighted imaging images located it in the prostatectomy bed or in prostate gland.
- Biparametric magnetic resonance imaging-based prostate magnetic resonance imaging for local recurrence reporting distinguishes 4 categories from 1 to 4 and assigns to each one the probability of recurrence diagnosis.

## Introduction

Biochemical recurrence (BCR) of prostate cancer (PCa) occurs within 10 years in 30-50% of patients receiving radiation therapy (RT) and in 20-40% of patients undergoing radical prostatectomy (RP) .^1,2^ The BCR in patients after RT is confirmed when the prostate specific antingen (PSA) value is ≥ Nadir + 2 ng/mL^3,4^ and after RP when the PSA increases and reaches a threshold of 0.2 ng/mL, twice consecutively after surgery^5^ without evidence of disease on imaging.

Although the role of dynamic contrast enhanced (DCE) remains unclear, the multiparametric MRI (mpMRI) is reported as an useful method in the detection of local recurrence after RT and after RP.^6,7^ Recently, for detecting prostate cancer local recurrence (PCLR) in patients with BCR after RT or RP, the Prostate Imaging for Recurrence Reporting (PI-RR) has been developed to standardize mpMRI acquisition, interpretation, and reporting.^8^ PI-RR which showed promising results similar to those of previous, proposed mpMRI studies of a 5-point scoring system for RT and RP and for each one it indicated the actual probability of PCLR diagnosis.^9,10-14^

To date, noncontrast or bpMRI (T2-weighted imaging [T2WI], diffusion-weighted imaging [DWI]/apparent diffusion coefficient [ADC]) using a dedicated simplified Prostate Imaging Reporting and Data System (S-PI-RADS) in the detection of PCa is reported.^15,16^

However, in the detection of PCLR after treatment, sporadic studies have been proposed using PSA and bpMRI as potential diagnostic tool.^17^

Unlike PCa, the PCLR after RP and RT in the prostatectomy bed or in prostate gland, respectively, is characterized by an increase in cell density that can be detected on DWI/ADC as a restricted diffusion area.^6,17-21^

After RT, dynamic DCE added to T2WI and DWI does not significantly improve the detection of PCLR.^22^ After RP, postoperative changes may result in low specificity of DCE in detection of tumor recurrence and the enhancement may be reduced in patients undergoing androgen deprivation therapy (ADT).^23,24^

In the detection of PCa after RT, the DWI unlike DCE can be performed after completing RT^11^ and in combination with T2WI has higher diagnostic accuracy and inter-reader agreement.^6^ After RP, DWI shows 100% specificity in detecting nodules at ≥1 cm.^25^ In general, after treatment in favor of bpMRI, the elimination of risks associated with intravenous administration of gadolinium-based contrast media, reduced costs, and shorter scan times should be considered.

Despite the above advantages, the lack of a dedicated standardized scoring system in the detection of PCLR after RT or RP limits the adoption of bpMRI. For this reason, a PI-RRADS to standardize bpMRI acquisition, interpretation, and reporting has been proposed, to facilitate multidisciplinary cooperation.^26^

Our aim is to assess the added value of bpMRI in the detection of PCLR in patients with BCR after RT and RP and to propose PI-RRADS in the probability of recurrence diagnosis.

## Material and Methods

The study was approved by the Ethic Committee of Umbria, Italy (Approval No: CER 4338) and informed consent was waived.

We retrospectively analyzed 86 patients with BCR after RT (n = 48) and after RP (n = 38) referred at the Radiotherapy Oncology Centre Santa Maria Hospital, Terni, from March 2018 and November 2021 and at the Department of Medicine and Surgery of the Santa Maria della Misericordia Hospital, University of Perugia, - from January 2016 and June 2022.

Before RT and RP, Gleason score (GS) ranged from 6 to 8 and 6 to 9, respectively.

The median PSA at relapse disease in the setting of patients after RT was 3.7 ng/mL (range, 2.3-4.2 ng/mL) and after RP 1.15 ng/mL (range 0.20-12.94 ng/mL).

The mean BCR time of disease relapse after RT and RP was 38 months (range 21-76 months) and 94.5 months (range 6-204 months), respectively.

Out of 86 patients, 55 (20 after RT and 35 after RP), without evidence of regional or distant metastasis at choline or gallium-68-PSMA positron emission tomography/computed tomography (PET/CT) or bone scan) at the time of bpMRI, were included in the study.

Out of 48 patients after RT, 28 were excluded from the study [patients with pelvic lymph nodes relapse (n = 15), patients with bone metastases (n = 8), and patients with lymph nodes and bone progression of the tumor (n = 5)]. Out of 38 patients after RP, 3 patients were excluded from the study (2 patients with bone metastases, and 1 patient with lymph node and bone metastases). None of the patients included in the study had general MRI contraindication, nor they were on hormonal treatment during the last 3 months nor did they have clip artifacts in the prostatectomy fossa after RT.

Our study includes 55 patients after RT (n = 20) and after RP (n = 35).

To compare PSA and bpMRI results, a group of 15 patients with PSA values <0.20 ng/mL, not consistent with BCR, was was also considered.

### Prostate Biparametric Magnetic Resonance Imaging Protocol

BpMRI at 3T (Philips Achieva, Medical System and Siemens Magnetom Verio) without endorectal coil (ERC) pulse sequences for PCLR workup included multiplanar T2WI, axial DWI using *b* values of 0-1500 s/mm^2^ with ADC map calculation, axial pelvic T1W with fat suppression, and T2W SPIR sequences. Scanning at 1.5T with optimal protocols would also result in diagnostic image quality. Post-treatment prostate bpMRI at 3T protocol without ERC is reported in Table 1.

### Images Analysis

BpMRI exams were independently analyzed by 3 radiologists (MS, ADB, and FMM) with more than 10 years of experience in prostate MRI and the discrepancy reviewed in consensus.

**Figure 1.**
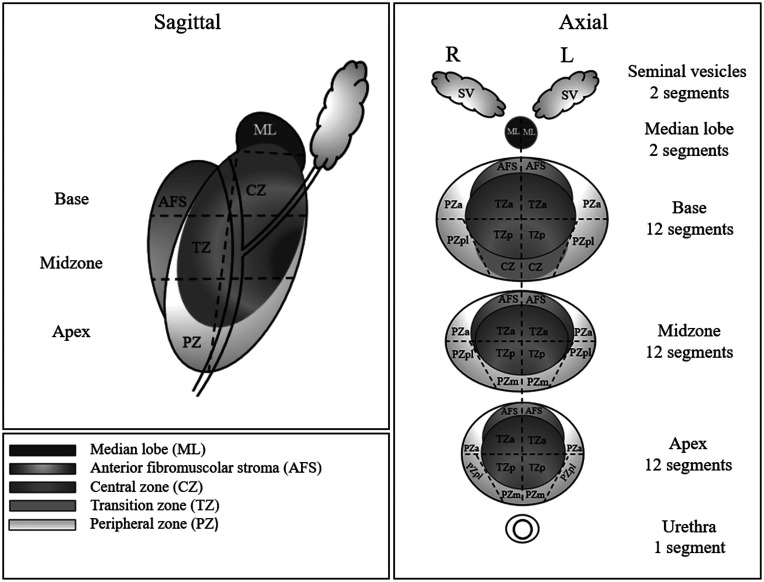
41-sector map of the prostate. Illustration recreated according to Scialpi et al.^11^ Median lobe (ML), anterior fibromuscolar stroma (AFS), central zone (CZ), transition zone (TZ), and peripheral zone (PZ).

The bpMRI sequences were analyzed in a standardized way including the following steps:

1. axial DWI with high *b* values and corresponding ADC maps for detection of local recurrence in the irradiated prostate gland (peripheral zone [PZ] and transition zone [TZ]) and in the prostatectomy fossa after RT and RP, respectively;
2. triplanar T2WI for the localization of the lesions detected on DWI/ADC. After RT, the lesion was localized on T2WI 41-sector map of the prostate^29^ (Figure 1); the volume of the prostate which appeared smaller compared to that of the pretreatment was measured by ellipsoidal formula (width × height × length × 0.52); for lesions in the PZ in T2WI the extracapsular extension (ECE) which was suspected in the presence of capsular overshoot, swelling or contact extension was assessed.

**Figure 2.**
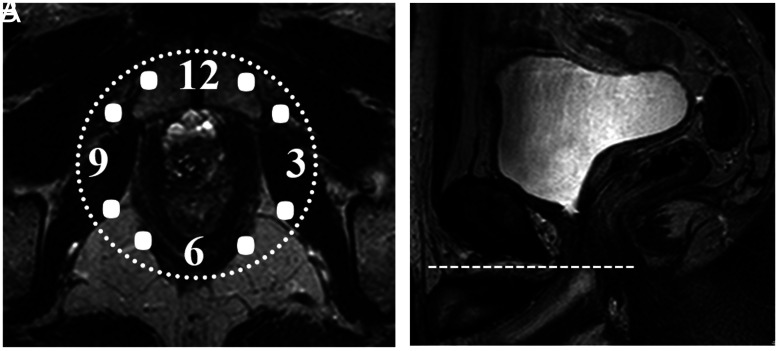
Schemes for localization of recurrence in the prostatectomy bed after radical prostatectomy. (A) Axial T2W images vesico-urethral clock and (B) T2W sagittal images on which the lesion is localized with respect to a plane passing through the lower margin of the pubic symphysis.

After RP, the lesion in the prostatectomy bed (vesico-urethral anastomosis, retrovesical space, bladder neck, near the seminal vesicles bed, or adjacent to the vas deferens)^17,20^ was localized using the landmarks as vesico-urethral clock on the axial T2WI and the distance of the lesion from the lower margin of the pubic symphysis on the sagittal T2WI) after RT (Figure 2).

The main criteria to confirm the presence of local recurrence on bpMRI were:

1. presence of any area of hyper-/moderate or marked hypointensity on DWI at high b-values/ADC map and corresponding slightly hypointense or intermediate signal intensity on T2WI in both the prostate after RT and prostatectomy bed after RP;
2. persistent significant reduction of PSA value in patient with bpMRI PCLR both prostatectomy and in patients treated with salvage RT; the response after RT was assessed with PSA evaluation scheduled every 3 months during the first year and then every 6 months. After surgery a PSA value > 0.2 ng/ml was considered a BCR, on the contrary a rise by 2 ng/mL or more above the nadir PSA was the standard definition for biochemical failure after external RT with or without concomitant hormotherapy;^3^
3. decrease and stability of PSA levels in patient with bpMRI PCLR who were treated with ADT at 24 months follow-up.

Criteria for local recurrence presence at bpMRI after RT and RP in patients with BCR are reported in Table 2.

Based on the above bpMRI criteria, 4 PI-RRADS categories from 1 to 4 were distinguished and the probability of recurrence diagnosis was assigned to each as follows:

1. PI-RRADS category 1: very low probability of recurrence;
2. PI-RRADS category 2: low probability of recurrence;
3. PI-RRADS category 3: high probability of recurrence;
4. PI-RRADS category 4: very high probability of recurrence.

PI-RRADS assessment categories and probability of recurrence are summarized in Table 3 and prostate bpMRI structured report using PI-RRADS scheme is shown in Table 4.

### Statistical Analysis

Statistical Package for Social Sciences version 23.0 (IBM SPSS Corp.; Armonk, NY USA) and MEDCALC softwares were used to perform statistical analyses. In the descriptive statistics, data were expressed as counts and percentages. For the analyses, PSA and bpMRI results were categorized in 2 levels (0 and 1), which respectively included all values < and ≥ the cut-off value of 0.2 ng/mL, for PSA (PSA_encod_), and all cases without and with restriction, for bpMRI (bpMRI_encod_). The agreement between biochemical and MRI approach results was quantified measuring the Cohen’s coefficient *k*, while their correlation using the Fisher exact test.

The diagnostic accuracy of bpMRI_encod_ included measures of sensitivity, specificity, positive predictive value (PPV), negative predictive value (NPV), false-positive value, false-negative value, and total diagnostic accuracy value (percentage of cases correctly diagnosed).

The ability of bpMRI_encod_ and other variables (i.e., time for recurrence, lesion diameter, S-PI-RADS category and GS) in predicting PCLR was tested in a bi- and a multivariable prediction model using a bivariate and multivariate analysis. For the multivariate model, all variables that showed a corrected *P* (Pc) value ≤ .25 could be included as explanatory. To avoid multicollinearity problems, predictors in strong correlation with other explanatory variables were excluded. The respective odds ratios (ORs) with 95% CIs were calculated. Logistic regression was then complemented by a predictive accuracy test that was quantified as the area under the receiver operating characteristic (ROC) curve. Statistical difference between the areas under the ROC curves was also assessed and, when considering PSA_encod_, the standard error of the mean (SEM) was conventionally set equal to10^-6^.

## Results

On 55 patients enrolled with BCR, bpMRI detected 20 lesions in 20 out of 20 patients after RT, and 48 in 31 out of 35 individuals after RP.

**Figure 3.**
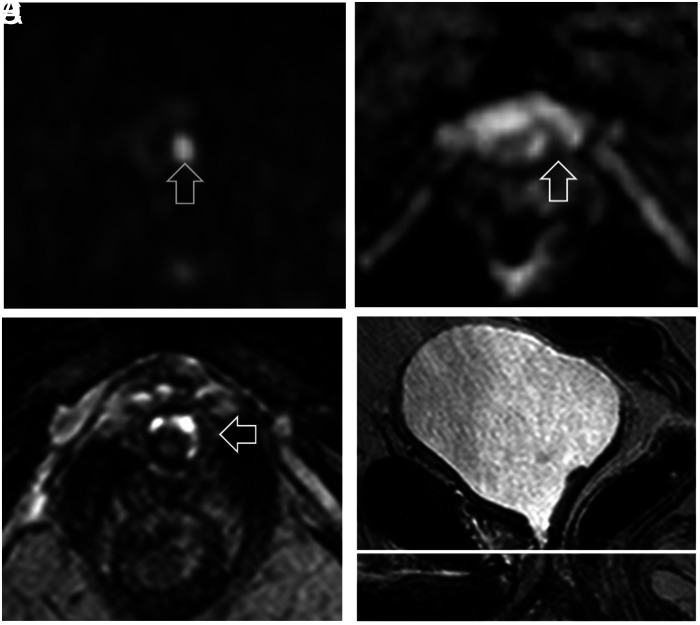
PI-RRADS category 3 based on biparametric MRI, 5 years after RP for prostate adenocarcinoma Gleason 7 score, in 87-year-old patient with PSA increased by 0.67 ng/mL. The lesion appears hyperintense on DWI at high *b* value (arrow in A), moderately hypointense on ADC map (arrow in B) and slightly hypointense on T2W images (arrow in C). The lesion is located at perianostomosis in the left at 2-4 clock cranially 10 mm above a through plane passing through the lower margin of the pubic symphysis (arrow in D).

**Figure 4.**
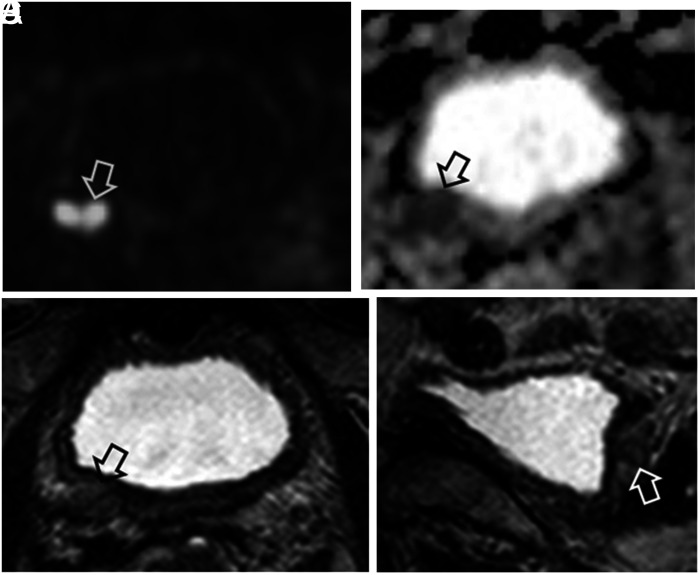
PI-RRADS category 4 based on biparametric MRI, 8 years after RP for prostate adenocarcinoma Gleason 7 score, in 74-year-old patient with PSA increased by 2.86 ng/mL. The lesion detected as hypointense on DWI at high *b* value (arrow in A) and inhomogeneous moderately/markedly hypointense on ADC map (arrow in B) and inhomogeneous on T2W images (arrow in C). The lesion is located at the level of the right posterior wall of the bladder cranially 15 mm above a plane passing through the lower margin of the pubic symphysis (arrow in D).

In the setting of patients after RT, the 20 lesions ranged in size from 5 to 15 mm (mean 11 mm) and were localized in the PZ (n = 17) and in the TZ (n = 3). Five out of 20 lesions (25%) were assigned to PI-RRADS category 3, and 15 out of 20 (75%) to PI-RRADS category 4 (Figures 3 and 4). All patients had a decrease in serum PSA level in the first 6 months; 3 patients had a progressive increase in serum PSA at the 9, 12, and 15 months, respectively.

In the setting of patients after RP, the 48 lesions in 31 out of 35 patients ranged in size from 5 to 2.1 cm (mean 1.11 cm). Lesions were located in the bladder neck/periurethral anastomosis site (n = 41), bladder floor (n = 4), and retroischiatic (n = 3).

**Figure 5.**
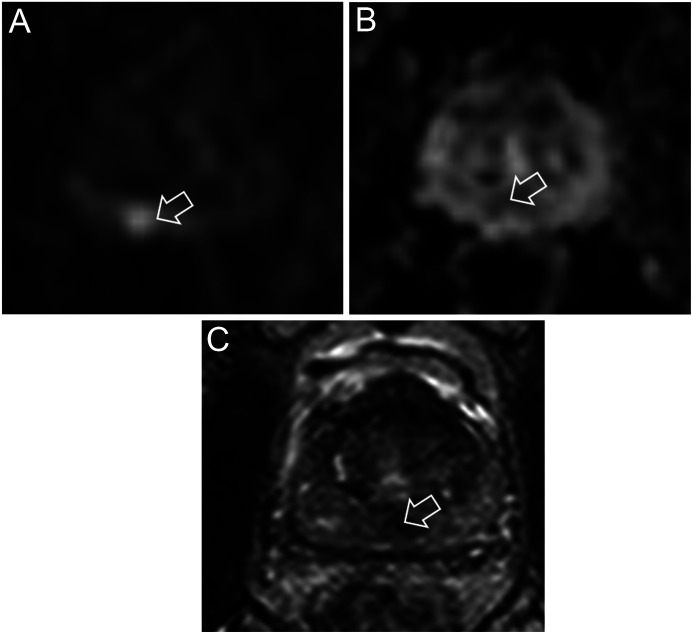
PI-RRADS category 3 based on biparametric MRI, 13 years after RT for prostate adenocarcinoma Gleason score 7, in 77-year-old patient with increased PSA 2.09 ng/mL The lesion detected as hyperintense on DWI at high *b* values (arrow in A) and moderately hypointense on ADC map (arrow in B) and hypointense inhomogeneous on axial T2W imaging (arrow in C) occurs at the site of the tumor of origin in the base in the right median segment of the peripheral zone.

**Figure 6.**
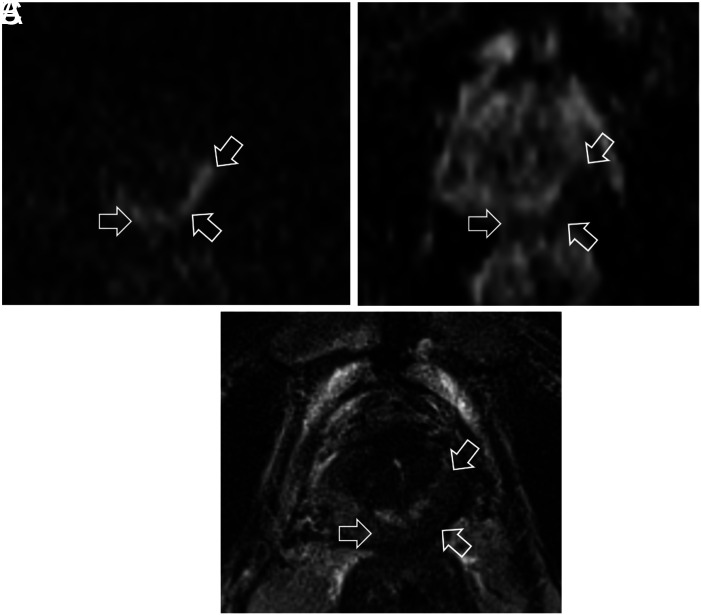
PI-RRADS category 4 based on biparametric MRI, 2 years after RT for prostate adenocarcinoma Gleason score 8, in 75-year-old patient with increased PSA 2.4 ng/mL The lesion detected as hyperintense on DWI at high *b* values (arrow in A) and markedly hypointense on ADC map (arrow in B) and hypointense inhomogeneous on axial T2W imaging (arrow in C) occurs at the site of the tumor of origin in the mid-gland in the left lateral segment of the peripheral zone.

No lesions were detected in 4 patients (11.4%) who were assigned to PI-RRADS categories 1 and 2 (negative). Out of 48 lesions, detected in 31 patients, 21 (43.8%) were categorized as PI-RRADS 3, and 27 (56.3%) as PI-RRADS 4 (positive) (Figures 5 and 6).

All patients had low and stable PSA (median PSA 0.63 ng/mL, range from 0.01-5.07 ng/mL) at 12 months after treatment; 1 patient showed stable PSA at follow-up of 1 year.

The Cohen’s coefficient *k* (0 .810, *P* < .001) demonstrated an excellent agreement between bpMRIencod and BCR data. This evidence was also sustained by results of correlation, performed with Fisher exact test (*P* < .001), and measures of diagnostic accuracy. The sensitivity, specificity, PPV, NPV, false-positive, false-negative, and total diagnostic accuracy values of bpMRIencod were 96.15%, 86.7%, 97.4%, 81.25%, 13.3%, 3.8%, and 94.6%, respectively.

Results of bivariate and multivariate analyses, settled to test the ability of bpMRIencod and other variables (i.e., time for recurrence, lesion diameter, S-PI-RADS category, and GS) in determining PCa recurrence were resumed in Table 5. In the logistic regression analyses, bpMRI_encod_ alone was recognized as the best predictor in determining PCLR.

## Discussion

Detection of PCLR is extremely important for predicting prognosis, guiding treatment decisions, and successful salvage therapy.

PSA remains the most used test to assess the response after therapy including the RT^28^; however, the PSA values alone may not necessarily lead to clinically evident metastatic disease^29^ and significantly delay treatment decisions.

Gallium-68-PSMA PET/CT shows promising diagnostic tool in PCa BCR, however to date there are no definitive results on its diagnostic accuracy in detecting PCLRs after RT and RP. In a study by Carvalho et al^30^ in RP (pre-PET PSA ≥ 0.8 ng/mL) and RT (pre-PET PSA ≥ 2.3 ng/mL) patients, gallium PSMA PET/CT detected pelvic relapse in 31.3% and 63.6%, respectively. In the setting of post-RP patients, urinary excretion of PSMA choline or gallium obscures local recurrence in the prostatic fossa due to the superimposed activity in the urinary bladder.^31^

In the setting of post-RT patients, mpMRI is an accurate method in the detection PCLR; a median sensitivity of 84% and specificity of 92% for the detection of PCLR is reported.^10^ After RT, sensitivities and specificities ranging from 72% to 100% and 85% to 100%, respectively^11-14^

Recently, PI-RR assessment to standardize mpMRI acquisition and reporting and to provide a reproducible tool for detecting PCLR in patients with BCR after RT or RP was developed.^8^ PI-RRprovides 5-point scoring system for RT and, RP and for each one, the actual probability of recurrence.

The PI-RR system created via consensus through a combination of face-to-face and online discussions is not based on high-level scientific evidence; it considers DCE to be the dominant sequence in detecting PCLR treatment, it does not indicate landmarks for recurrence location after RP that are crucial in selecting the best treatment modality. In addition the generalizability of results to the community setting is limited by the experience of radiologists.

Similar to S-PI-RADS based on bpMRI, we recently proposed a novel dedicate reporting system, named PI-RRADS,26, which establishes minimum acceptable MRI parameters for the detection PCLR after RT and RP.

BpMRI-based PI-RRADS assigns to DWI/ADC the main role in detecting recurrence in the prostate gland both in PZ and in TZ after RT and in prostatectomy bed after RP. We assume that, as for PCa, PCLR is histologically characterized by a cell density that determines areas of reduction of water diffusion and moderate or marked hypointensity on the ADC maps. PI-RRADS provides 4 assessment categories and assigns to each one the probability of recurrence from very low to very high. PI-RRADS category 1 and 2 (negativity for recurrence in the prostate and in the prostatectomy bed after RT and RP, respectively) have very low and low probability for recurrence, respectively. Areas with moderate hypointensity in ADC map were assigned to PI-RRADS category 3 (high probability for recurrence) whereas those with marked hypointensity in ADC map were assigned to PI-RRADS category 4 (very high probability for recurrence).

ADC gray scale map by quantitative analysis may improve the discrimination between category 3 and category 4 making the system reliable and reproducible.^26^

After RT, T2WI allows the detection of suspicious areas in the prostate using a 41-sector map and assesses the capsular involvement. After RP, T2WI is able to localize the lesion using as landmarks the vesico-urethral clock on axial planes and the inferior margin of pubic symphysis on sagittal planes. An accurate location of the lesion is critical to guide treatment decisions and to the success of salvage therapy.

Our preliminary study has some limitations. First, its retrospective nature including a limited number of patients. A prospective study in a large series is needed to confirm our findings. Second, patients with BCR irradiated after RP or re-irradiated after RT underwent ADT. In these patients, distant recurrences were excluded with choline or gallium PSMA PET/CT or bone scintigraphy; however, it was not possible to state with certainty the change or disappearance of local recurrence because the bpMRI was not repeated after treatment. On the contrary, the zeroing of PSA level following local treatment alone [radiotherapy (RT)] is an indirect sign of local recurrence in patient with BCR following radiation therapy. All patients who received treatment showed decrease of PSA in at least 24 months. Third, our study does not aim to define the sensitivity of bpMRI in detecting PCLR but to evaluate the added value of bpMRI by defining the probability of disease and the lesion localization in the prostate or prostatectomy bed to decide the salvage RT.

In conclusion, in men with BCR,^32^ when choline or gallium-68-PSMA PET/CT are not consistent with PCLR, prostate bpMRI in association to PSA and the GS of the previous biopsy or prostatectomy, is a potential valuable tool to detect and localize PCLR after RT and RP. The ­proposed bp-MRI-based PI-RRADS indicates the probability categories for PCLR and can facilitate collaboration between radiologist and ­clinician. A multi-institutional prospective study is critical to assess the effectiveness of the PI-RRADS and the contribution of the bpMRI in the detection and categorization of PCLR after RT and RP.

## Figures and Tables

**Table 1.** Prostate Biparametric Magnetic Resonance Imaging Protocol: Sequence Parameters at 3T

Sequences/Plane	TR/TE (ms)	Slice Thickness (mm)	Gap	*b* values (s/mm^2^)	NEX	FOV (mm)	Matrix
DWI/axial	3.690/67	2	0	0-1500	3	Large	124 × 100
T2W/TSETriplanar	8380-12752/90	2	0	–	2	180 × 180	188 × 166
T1W-THRIVE/axial	3.0/1.5	1.5	–	–	1	Large	252 × 198
T2W SPIR	7000/120	2	0	–	1	Large	260 x 426

ADC maps were generated from DWI images with *b* values of 0, 500, 1000, and 1500 s/mm^2^.

DWI, diffusion-weighted imaging; FOV, field of view; NEX, number of excitations; SPIR, spectral presaturation with inversion recovery; T1W, T1-weighted; T2W, T2-weighted; TE, echo time; THRIVE, T1 high-resolution isotropic volume excitation; TR, repetition time; TSE, turbo spin-echo.

**Table 2.** Criteria for Local Recurrence Confirmation at bpMRI After Radiation Therapy and Radical Prostatectomy in Patient with Biochemical Recurrence

Treatment	Patients/Age	PSA at Relapse Disease	BpMRI at Relapse Disease	Nuclear Imaging at Relapse Disease	PSA Postreatment
Radiation therapy (RT)	20 patients, age range 75-81 years (mean 76 years)	Range 5.3-12.9 ng/mL (mean 7.6 ng/mL)	Positive (20/20)	Negative (n = 20)	Range 2.3-4.2 ng/mL (mean 3.7 ng/mL)^*^
Radical prostatectomy (RP)	35 patients, age range 52-85 years (mean 74.6 years)	Range 0.20-12.94 ng/mL (1.15 ng/mL)	Positive (31//35)	Negative (n = 35)	Range 0.01-5.07 ng/mL (mean 0.63 ng/mL)^**^

^*^In the first 6 months, all 20 patients had a decrease in serum PSA level; 3 of the 20 patients had a progressive increase in serum PSA at months 9, 12, and 15, respectively.

^**^All patients had low and stable PSA levels (median PSA values of 0.63 ng/mL, range from 0.01 to 5.07 ng/mL) at 12 months after treatment; 1 patient had stable PSA values at 1-year follow-up.

**Table 3.** PI-RRADS bpMRI-Based Assessment Categories

PI-RRADS					
Category	DWI/ADC		T2WI		Probability
	RT	RP	RT	RP	
1	No abnormality	No abnormality	Homogeneous intermediate signal intensity (normal)	No abnormality	Very low
2	No restricted diffusion	No restricted diffusion	Focal or diffuse moderate hypointensity or nodule	Diffuse thickening or nodule at vesicourethral anastomosis or nodule or mass in the prostatectomy bed	Low
3	Focal rounded, lenticular or irregular or mass-like hyperintensity on DWI at high *b* values and moderate homogeneous hypointensity on ADC map	Lobulated, curvilinear, or semi-circumferential mass, nodular or plaque-like or irregular hyperintensity on DWI at high *b* values and moderate homogeneous hypointensity on ADC map	Moderate hypointensity compared to the irradiated prostatic tissue in the corresponding site of DWI/ADC	Intermediate signal intensity or slightly hyperintensity compared to pelvic muscle in the corresponding site of DWI/ADC	High
4	Focal rounded, lenticular or irregular or mass-like hyperintensity on DWI at high *b* values and marked homogeneous or inhomogeneous hypointensity on ADC map	Lobulated, curvilinear, or semi-circumferential mass, nodular or plaque-like or irregular hyperintensity on DWI at high *b* values and marked homogeneous or inhomogeneous hypointensity on ADC map	Moderate hypointensity compared to the irradiated prostatic tissue in the corresponding site of DWI/ADC	Intermediate signal intensity or slightly hyperintensity compared to pelvic muscle in the corresponding site of DWI/ADC	Very high

In men with suspected biochemical recurrence (BCR) (Ref. 32)after RP and RT, prostate bpMRI may be considered in association to PSA and the Gleason score of the ­previous biopsy or prostatectomy and when whole-body imaging (e.g., PET/CT with prostate-specific membrane antigen positron emission tomography) is not consistent with recurrent disease.

ADC, apparent diffusion coefficient; bpMRI, biparametric magnetic resonance imaging; DWI, diffusion-weighted imaging; PI-RRADS, Prostate Imaging for Local Recurrence Reporting and Data System; RP; radical prostatectomy; RT, radiotherapy; T2WI, T2-weighted imaging.

**Table 4.** PI-RRADS Reporting

PI-RRADS Reporting	
Clinical notes	Absolute and doubling time of PSA value. Histological information about Gleason score before treatment. Schedule of post-treatment (RT or RP). Relevant clinical history of PCa.
Technical details	Patient preparation, MR scanner field strength, quality of DWI and T2WI. For DWI or T2WI of inadequate quality, the causes of the problems should be mentioned and, if possible, the solutions performed should be reported.
Findings	In patient with RT, the prostate volume, reduced respect to pre-treatment value, calculated on axial and sagittal T2WI using the ellipsoidal formula (maximum anterior posterior diameter × maximum transverse diameter × maximum longitudinal diameter × 0.52) is reported. Morphology, size, and signal intensity on DWI/ADC is indicated. Location on 41-sector map and for lesion in PZ, border configuration, possible EPE, and infiltration of the neurovascular bundle and seminal vesicles as well as infiltration of adjacent organs T2WI are reported in this section. In patient with RP, morphology, size, and signal intensity of lesion on DWI/ADC is indicated. Lesion localization on T2WI in the corresponding site of DWI/ADC at retrovesical space, bladder neck, near the seminal vesicles bed, or adjacent to the vas deferens; for lesion localization in the prostatectomy bed (vesico-urethral anastomosis), the vesico-urethral clock on the axial T2W and the distance of the lesion from the lower margin of the pubic symphysis on the sagittal T2W are used as landmarks. Findings are described according to PI-RRADS. After RT and RP, 4 categories of PI-RRADS from 1 to 4 are described and for each the probability of recurrence is indicated.
Additional findings	Presence or absence of possible lymph node metastases in the entire pelvis are described and localization, number and size are mentioned. Additionally, if present, bone metastases are described. Finally, additional findings in the scanned volume are mentioned.

The biochemical relapse of prostate cancer after local therapy was diagnosed according to EAU-ESTRO-SOG guidelines. Relapse after local therapy is defined by a rising PSA level > 0.2 ng/mL following RP and >2 ng/mL above the nadir after RT^20-22^.

PI-RRADS, Prostate Imaging for Local Recurrence Reporting and Data System.

**Table 5.** ROC Curves of Individual Predictor Variables in Bivariate and Multivariate Analysis

	ROC Curve	Bivariate Analysis	Multivariate Analysis
	AUC (95% CI)	OR (95% CI); *P*	OR (95% CI); *P*
bpMRI_encod_	0.914 (0.810-1.0)	162.5 (24.7-1068); *P *< .001	31.9 (1.6-615.4); *P *= .02
Time for recurrence^a^	0.618 (0.494-0.724)	1.02 (1.0-1.045); *P *= .053	–
Lesion size	0.959 (0.882-1.0)	2.0 (1.45-2.7); *P *< .001	1.6 (1.2-2.29); *P* = .003
S-PI-RADS category^b^	0.886/(0.759-1.0)	39 (7.3-208); *P* < .001	–
GS^c^	0.516 (0.373-0.658)	1.2 (0.81-2.09); *P* = .254	–
Multivariate model: mpMRI_encod_, lesion size	0.959 (0.889-1.0)		

Results demonstrated that in bivariate logistic regression models, bpMRIencod (*P* < .001), time for recurrence (*P* = .053), lesion dimension (*P* < .001), and S-PI-RADS category (*P* < .001) were significantly associated with PCa recurrence, while GS (*P* = .245) was not.

bpMRIencod (OR = 162.5) was the best predictor. Comparing the areas under the ROC curves, no statistical differences were seen between bpMRIencod and lesion dimension, and also between these 2 variables versus the best multivariate logistic model, which included them both. Remarkably, the areas under the ROC curves of bpMRIencod (0.914, SEM 0.053) and PSAencod (1.0, SEM 10-6) were not statistically different.

^a^Not included in the multivariate model because of OR value ≈ 1.

^b^Not included in the multivariate model because of the correlation with mpMRI_encod_.

^c^Not included in the multivariate model, *P* ≥ .25.

AUC, area under the curve; GS, Gleason score; OR, odds ratio; ROC, receiver operating characteristic.
